# A risk score model based on TGF-β pathway-related genes predicts survival, tumor microenvironment and immunotherapy for liver hepatocellular carcinoma

**DOI:** 10.1186/s12953-022-00192-4

**Published:** 2022-06-22

**Authors:** Jingsheng Liao, Qi Liu, Jingtang Chen, Zhibin Lu, Huiting Mo, Jun Jia

**Affiliations:** 1 Department of Medical Oncology, Affiliated Dongguan Hospital, Southern Medical University, 78 Wandao Road, Dongguan City, 523000 Guangdong Province China; 2 Department of Medical Oncology, Dongguan Institute of Clinical Cancer Research, 78 Wandao Road, Dongguan City, 523000 Guangdong Province China

**Keywords:** TGF-β signaling pathway, Liver hepatocellular carcinoma, Tumor microenvironment, Prognosis, immunotherapy

## Abstract

**Background:**

Transforming growth factor-beta (TGF-β) signal is an important pathway involved in all stages of liver hepatocellular carcinoma (LIHC) initiation and progression. Therefore, targeting TGF- β pathway may be a potential therapeutic strategy for LIHC. Prediction of patients’ tumor cells response requires effective biomarkers.

**Methods:**

From 54 TGF-β-related genes, this research determined the genes showing the greatest relation to LIHC prognosis, and developed a risk score model with 8 TGF-β-related genes. The model divided LIHC patients from different datasets and platforms into low- and high-risk groups. Multivariate Cox regression analysis confirmed that the model was an independent prognostic factor for LIHC. The differences in genetic mutation, immune cell infiltration, biological pathway, response to immunotherapy or chemotherapy, and tumor microenvironment in LIHC samples showing different risks were analyzed.

**Results:**

Compared with low-risk group, in the training set and test set, high-risk group showed shorter survival, lower stromal score and higher M0 macrophages scores, regulatory T cells (Tregs), helper follicular T cells. Moreover, high-risk samples showed higher sensitivity to cisplatin, imatinib, sorafenib and salubrinal and pyrimethamine. High-risk group demonstrated a significantly higher Tumor Immune Dysfunction and Exclusion (TIDE) score, but would significantly benefit less from taking immunotherapy and was less likely to respond to immune checkpoint inhibitors.

**Conclusions:**

In general, this work provided a risk scoring model based on 8 TGF-β pathway-related genes, which might be a new potential tool for predicting LIHC.

**Supplementary Information:**

The online version contains supplementary material available at 10.1186/s12953-022-00192-4.

## Background

In 2020, primary liver cancer was the third major cause resulting in cancer death worldwide, with morbidity and mortality rates 2–3 times higher in men than in women in most regions. Liver hepatocellular carcinoma (LIHC) is the most common primary liver cancer [1]. Although several staging systems, such as the Barcelona Clinic Liver Cancer (BCLC) system, Cancer of the Liver Italian Program (CLIP), TNM, Okuda, Japanese Integrated Staging (JIS) Score, have been developed to treat LIHC, but they all have some limitations that cannot be neglected [2]. The Barcelona Clinic Liver Cancer (BCLC) system, which was endorsed by European and American clinical practice guidelines, guides treatment choices and provides patients’ survival information [3]. Reasonable method for the diagnosis and treatment of LIHC has been developed, according to the BCLC stage. Application of these methods to comprehensive projects in high-risk populations has shown a great effectiveness in preventing LIHC and reducing overall mortality [4]. Development of new therapies and their combinations for treating adjuvant and intermediate-stage disease, discovery of biomarkers for therapeutic purpose could help treat LIHC [5].

Transforming growth factor-beta (TGF-β) signaling is present in initial liver injury to inflammation and fibrosis, to cirrhosis and LIHC [6]. During the development of LIHC, TGF- β has different effects on each stage of LIHC development. Early TGF- β inhibits liver tumorigenesis through inducing cell arrest and apoptosis, but once cells get rid of its inhibition, TGF- β develops the characteristics of migratory tumor initiation cells and promotes more advanced malignant progression via inducing cancer cell epithelial-mesenchymal transition (EMT), migration, invasion and final metastasis [7, 8]. Therefore, targeting TGF-β pathway might treat LIHC and help understand the potential pathogenetic mechanisms, with a special focus on the crosstalk between other signaling pathways and TGF-β [9]. Novel drugs that block TGF-β pathway have entered clinical evaluation, among which the most advanced is LY2157299, which has been confirmed in phase I studies to have antitumor activity in patients suffering from advanced LIHC through mainly affecting cancer cell migration and invasion [10]. In view of the dual role of TGF-β, there are still many factors hindering TGF-β inhibitors development, particularly patient selection, timing of treatment and predictive biomarkers [11].

In this study, TGF-β pathway-related genes from the Public Molecular Signature Database v7.4 (MSigDB) [12] were used to study the prognostic value of their expression profile in LIHC. Based on TGF-β pathway-related genes, a risk score model was designed for exploring the differences between different risk groups among immune cell infiltration, genetic changes, tumor microenvironment, response to immunotherapy or chemotherapy, function. A risk score model based on TGF-β pathway-related genes may be a promising tool for monitoring LIHC.

## Methods

### Acquisition of TGF-β pathway-related genes

A gene set within TGF-β pathway with suppressing, driving or mark function were obtained from MSigDB (v7.4). After removing duplicates, a total of 54 TGF-β pathway-related genes were retained for further analysis (Supplementary Table S1).

### Data acquisition of LIHC

LIHC data with clinical information, gene expression profiles and gene mutation data were acquired from three public databases, including The Cancer Genome Atlas database (TCGA, https://portal.gdc.cancer.gov/), Gene Expression Omnibus database (GEO, https://www.ncbi.nlm.nih.gov/geo/) and hepatocellular carcinoma database (HCCDB, http://lifeome.net/database/hccdb/home.html). TCGA-LIHC dataset including RNA sequencing (RNA-seq) data, gene mutation data, and clinical information was downloaded from TCGA database [13]. GSE10143 [14], GSE14520 [15] and GSE76427 [16] datasets containing gene expression profiles (microarray data) and clinical information were acquired from GEO database. ICGC-LIRI-JP dataset (named as ICGC (International Cancer Genome Consortium) in the following) containing RNA-seq data and clinical information was downloaded from HCCDB.

### Data preprocessing of LIHC

For LIHC samples in these datasets, samples without survival time, survival status or follow-up data were excluded. For RNA-seq data in TCGA-LIHC and ICGC datasets, Ensembl ID was converted to gene symbol. Averaged expression value was selected for one gene with multiple gene symbols. For gene expression profiles in GSE datasets, gene probes were converted to gene symbol. Probes targeting multiple genes were excluded. Averaged expression was used when multiple probes corresponding to one gene. Finally, after data preprocessing, 365 samples (130 censored and 235 uncensored, with the longest follow-up time of 10.0 years (only numbers were shown in the following)) from TCGA-LIHC dataset remained. 80 (32, 48, 15.6), 221 (85, 136, 5.5), and 115 (23, 92, 6.5) samples from GSE10143, GSE14520 and GSE76427 datasets remained, respectively. 203 samples (35, 138, 5.9) from ICGC dataset (Supplementary Table S2) remained. TCGA-LIHC dataset was set as a training dataset, and the other four datasets were independent dataset.

### Development of a prognostic gene signature

Firstly, univariate Cox regression analysis was conducted to determine prognosis-associated TGF-β pathway-related genes in TCGA-LIHC dataset. Least absolute shrinkage and selection operator (LASSO) Cox regression analysis in glmnet (v4.1) package [17] was performed to shrink the number of prognostic genes and construct an optimal prognostic model. LASSO allows a shrinkage estimation and construction of a simplified model with penalty function. The coefficients of prognostic genes were compressed by increasing lambda values. The optimal lambda value was confirmed by 95% confidence interval (CI) and examined by 10-fold cross-validation. Eight prognostic genes were identified with the optimal lambda value. Then the prognostic model was developed according to the expression of each gene and gene coefficients obtained from LASSO Cox regression. The risk model was defined as: risk score = Σ (coefficient i*expression i), where i indicated each gene.

Next, risk score for each sample was calculated, and samples were stratified into low- and high-risk groups according to the optimal cut-off value determined by surv_cutpoint function in survminer (v0.4.9) R package (https://cran.r-project.org/web/packages/survminer/index.html). Independent cut-off value of each dataset was calculated using the same algorithm. Kaplan-Meier survival analysis was conducted for measuring overall survival (OS) between high- and low-risk groups (log-rank test was performed). The effectiveness of the model was evaluated by receiver operating characteristic (ROC) analysis in timeROC (v0.4) R package [18]. Area under ROC curve (AUC) was calculated for assessing the performance of the prognostic model in each dataset.

### Gene set enrichment analysis (GSEA)

GSEA software (v4.2.0) developed by UC San Diego and Broad Institute (https://www.gsea-msigdb.org/gsea/index.jsp) was applied to further analyze enriched biological pathways of the two risk groups in TCGA-LIHC dataset [19]. A gene set of KEGG pathways “c2.cp.kegg.v7.4.symbols.gmt” was downloaded from MSigDB (https://www.gsea-msigdb.org/gsea/msigdb/index.jsp, v7.4) [12]. DESeq2 (v1.34) was employed to normalize RNA-seq data and produce a GSEA compatible “normalized counts” table in the gene cluster text (GCT) format [20]. Then normalized expression data grouped by risk groups was used as an input for conducting GSEA. Significantly enriched pathways with *P* < 0.05 were considered as significant.

### Gene mutation analysis for two risk groups

Gene mutation data in TCGA-LIHC dataset included tumor mutation burden (TMB), number of mutated genes, and single nucleotide variations (SNVs), which was already processed using mutect2 (v4.1.0.0) tool in The Genome Analysis Toolkit (GATK, https://gatk.broadinstitute.org/hc/en-us/articles/360037593851-Mutect2) by TCGA research group [21]. Student *t* test was applied for comparing TMB and number of mutated genes between high- and low-risk groups. *P* < 0.05 was considered as significant. *Chi*-square test was conducted for screening significantly mutated genes in the two risk groups (*P* < 0.05). The top 10 mutated genes were visualized by waterfall plot.

### Estimation of tumor microenvironment and immune cells

RNA-seq data of TCGA-LIHC were uploaded into Cell type Identification By Estimating Relative Subsets Of RNA Transcripts (CIBERSORT, https://cibersort.stanford.edu/) tool to evaluate the infiltration of 22 immune cells in the tumor microenvironment, including naïve and memory B cells, seven T cell types, plasma cells, myeloid subsets, and natural killer (NK) cells [22]. The scores of these immune cells between the two risk groups were compared by Wilcoxon test. In addition, Estimation of STromal and Immune cells in MAlignant Tumours using Expression data (ESTIMATE, v1.0.13) package was introduced to calculate ESTIMATE score, stromal score, and immune score, through indicating the fraction of immune and stromal cells in tumor samples using gene expression signatures [23].

### Prediction of immune/chemotherapy response

Based on modeling tumor immune evasion mechanism, the response to immunotherapy could be predicted by Tumor Immune Dysfunction and Exclusion (TIDE) tool [23]. To initially evaluate the response of two risk groups in the TCGA-LIHC dataset to immunotherapy, the TIDE algorithm was used. To further measure the response of each risk group to immunotherapy, we used subclass mapping (SubMap) analysis for comparing anti-programmed cell death ligand 1 (PD-L1) therapy response of the IMvigor210 dataset with that of TCGA-LIHC [24]. Furthermore, the half maximal inhibitory concentration (IC50) value of five chemotherapy or targeted drugs (imatinib, sorafenib, cisplatin, salubrinal and pyrimethamine) [25] for each sample in TCGA-LIHC dataset was evaluated by pRRophetic package [26].

### Statistical analysis

R software (v4.1) was employed for all statistical analysis. Survival R package (https://mran.microsoft.com/web/packages/survival/index.html) was utilized to perform Kaplan-Meier survival analysis, univariate and multivariate Cox regression analysis (log-rank test was conducted). The association of seven metagenes with the risk score was assessed by Pearson correlation analysis [27]. Differences between the two risk groups were analyzed by Wilcoxon test or student *t* test (indicated in the figure legends). If not specified, *P* < 0.05 was considered as significant.

## Results

### Development of the prognostic model

**Fig. 1:**
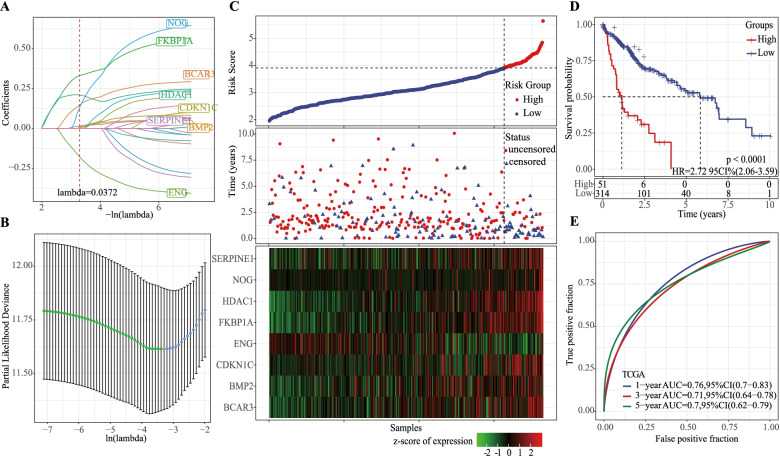
Development of the prognostic model with 8 TGF-β pathway-related gene in TCGA-LIHC dataset. **A** LASSO Cox regression analysis on 22 TGF-β pathway-related genes. The coefficients of genes closed to zero with the increasing lambda value. Dashed red line indicates the optimal lambda value = 0.0372 for constructing an optimal model with the least number of genes. **B** 95% CI of partial likelihood deviance under different lambda values evaluated by cross-validation. Red dot indicates the lambda value corresponding with the optimal model. The left (green dots) and right (blue dots) of the red dot indicate the lambda values with the decreasing and increasing number of genes respectively. **C** The distribution of samples with different survival status, and expression of eight prognostic genes for each sample ranking by risk score from low to high. Censored and uncensored indicate dead and alive status respectively. Horizontal axis indicates samples. Z-score of expression from green to red indicates low to high expression. **D** Kaplan-Meier survival curve of low- and high-risk groups classified by the prognostic model. Log-rank test was conducted. **E** ROC and AUC of the prognostic model in predicting 1-year, 3-year and 5-year OS. CI, confidence interval. HR, hazard ratio. ROC, receiver operation characteristic. AUC, area under ROC curve

The univariate Cox regression analysis of 54 TGF- β pathway-related gene was carried out with TCGA-LIHC dataset as training set to identify the prognostic TGF- β pathway-related genes in LIHC. The results showed that the expression levels of 22 TGF-β pathway-related genes were significantly correlated with the OS of LIHC. Then LASSO Cox regression analysis was performed to screen the most stable prognostic genes from the 22 TGF-β pathway-related genes. Partial likelihood deviance was the minimum when 8 variables were included in the model (Fig. 1A, B). Finally, TGF-β pathway risk score formula was constructed, according to 8 TGF-β pathway-related genes as risk score = 0.073*CDKN1C + 0.18*HDAC1 + 0.035*SERPINE1 + 0.068*BMP2–0.372*ENG + 0.392*FKBP1A + 0.481*NOG + 0.279*BCAR3. This formula was introduced to calculate the risk score of TCGA-LIHC samples and effectively distinguished the survival status of patients with different risks (Fig. 1**C**). 51 samples and 314 samples were classified into high- and low-risk groups, respectively, based on the optimal cut-off value (Supplementary Table S3). High-risk scored patients showed a significantly more unfavorable prognosis than patients with low-risk scores (Fig. 1D). For TCGA-LIHC dataset, AUC of risk score for predicting 1-year, 3-year, and 5-year OS was 0.76 0.71, and 0.70, respectively (Fig. 1E).

### Validation of the prognostic model in the GEO and ICGC datasets

**Fig. 2:**
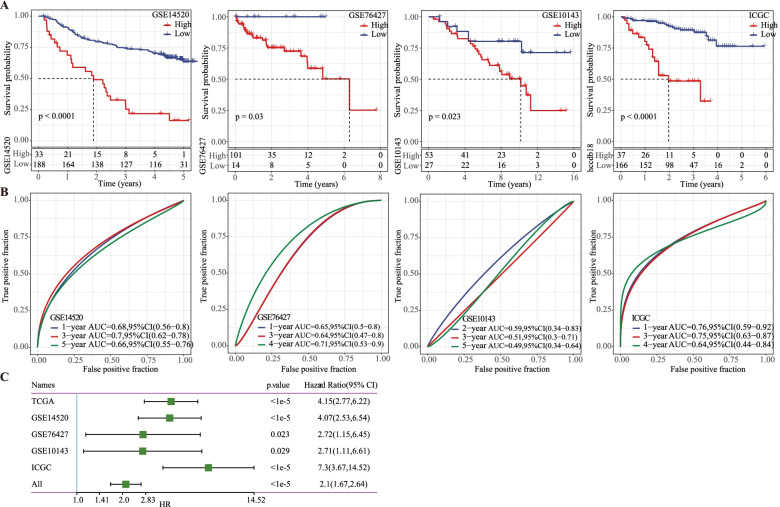
Verification of the prognostic model in GEO and ICGC datasets. **A** Kaplan-Meier survival curves of low- and high-risk groups in GSE14520, GSE76427, GSE10143, and ICGC datasets. **B** ROC analysis for evaluating the performance of the prognostic model in GSE14520, GSE76427, GSE10143, and ICGC datasets. **C** Univariate Cox regression analysis on the relation between risk score and prognosis in TCGA-LIHC, GSE14520, GSE76427, GSE10143 and ICGC datasets. Log-rank test was conducted. CI, confidence interval. HR, hazard ratio

To analyze the robustness of the model established based on TCGA-LIHC dataset, the risk scores of the samples were calculated by the same risk score formula. GSE14520, GSE76427, GSE10143 and ICGC were the external validation datasets. According to optimum cut-off values, LIHC samples in each dataset were grouped into two risk groups (Supplementary Table S3). Samples classified as high-risk were more likely to be in a death status, as demonstrated by the survival analysis of the samples in each dataset (Fig. 2A). The ROC curve showed that the prognostic model had a 3-year AUC values of 0.7 in GSE14520, 0.64 in GSE76427, 0.51 in GSE10143 and 0.75 in ICGC (Fig. 2B). Correlation of the OS of LIHC with the risk score of each dataset was also assessed by univariate Cox regression, and the risk score was found to be closely linked with OS in all the five datasets (Fig. 2C).

### Genetic variation and functional enrichment analysis of the prognostic risk model

**Fig. 3:**
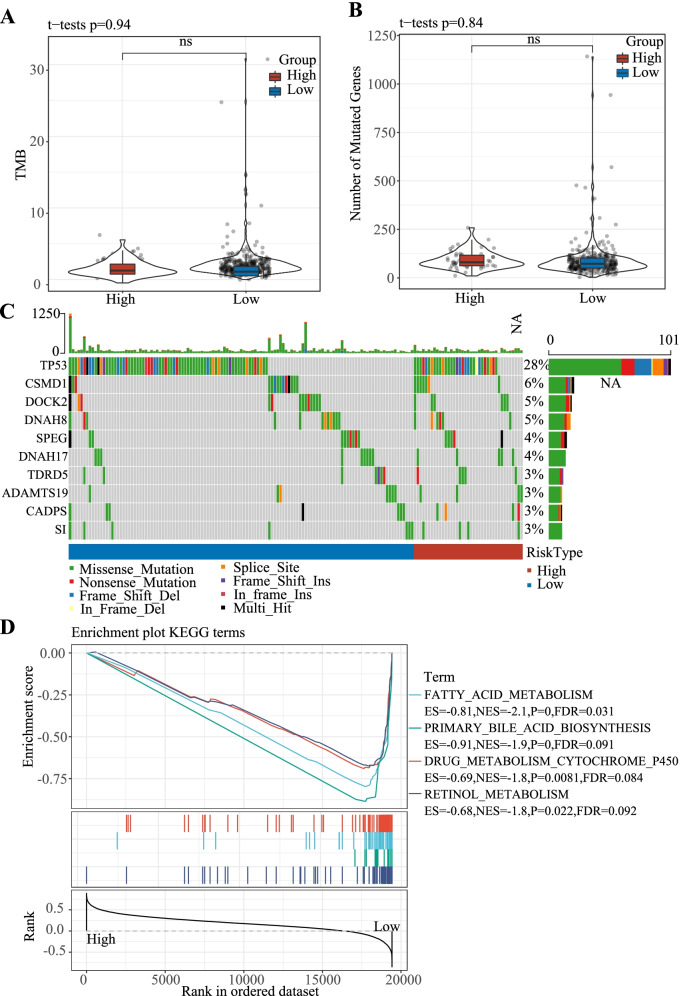
Gene mutation features and gene set enrichment analysis in TCGA-LIHC datasets. **A-B** Comparison of TMB (**A**) and number of mutated genes (**B**) between low- and high-risk groups. Student *t* test was conducted. **C** The waterfall diagram visualizing the top 10 mutated genes in the two risk groups (*P* < 0.05). *Chi*-square test was performed. **D** Enriched four KEGG pathways in low-risk group assessed by gene set enrichment analysis. TMB, tumor mutation burden. ES, enrichment score. NES, normalized enrichment score. FDR, false discovery rate. ns, no significance

Between the high- and low-risk groups, statistical analysis of TMB and nucleotide variation was not significantly different in TMB or nucleotide variation (Fig. 3A, B). In these two risk groups, single nucleotide variations in genes with the top 10 mutation frequencies were exhibited in Fig. 3C. The highest mutation frequency was present in TP53, the mutation rate of which was much higher than CSMD1 showing the second highest mutation frequency. GSEA on the two risk groups in TCGA-LIHC dataset revealed that metabolism-related pathways, including primary bile acid biosynthesis, fatty acid metabolism, retinol metabolism, and drug metabolism cytochrome P450, were significantly enriched in low-risk group, suggesting that the risk model can predict patient survival by reflecting LIHC metabolism (Fig. 3D).

### Correlation between risk score and clinical features

**Fig. 4:**
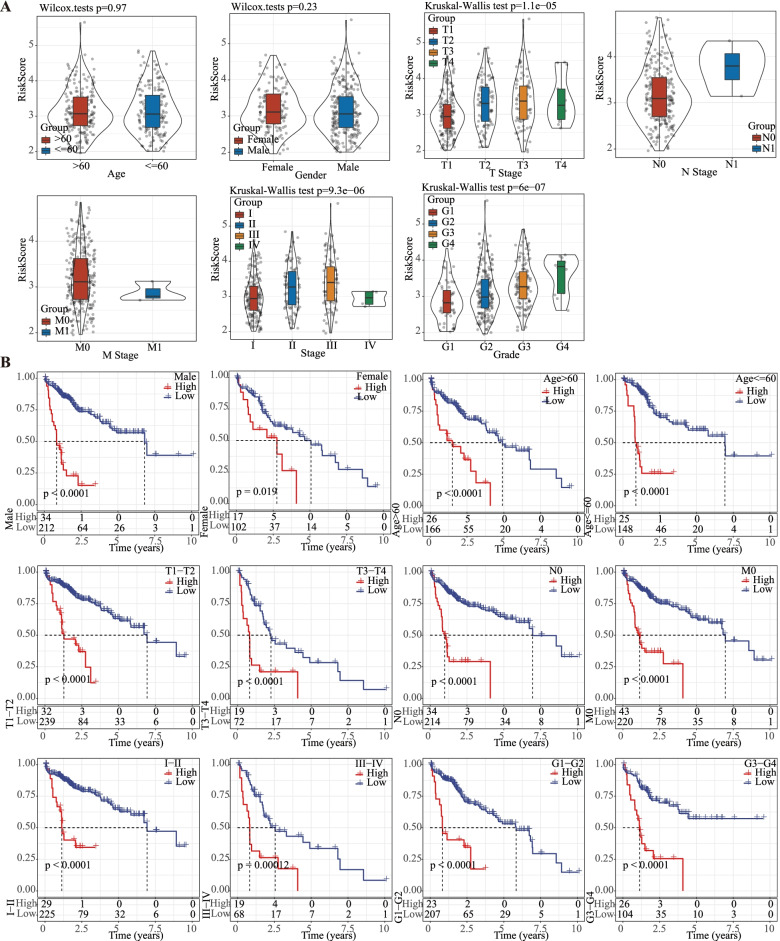
Correlation between risk score and clinical features in TCGA-LIHC dataset. **A** Comparison of risk score difference between different clinical features including ages, genders, T stage, N stage, M stage, AJCC stage I-IV, and grade. Wilcoxon test or Kruskal-Wallis test were conducted (No test was performed in N and M stages as insufficient samples). **B** Kaplan-Meier survival analysis on low- and high-risk groups classifying by different clinical features. Log-rank test was performed

**Fig. 5:**
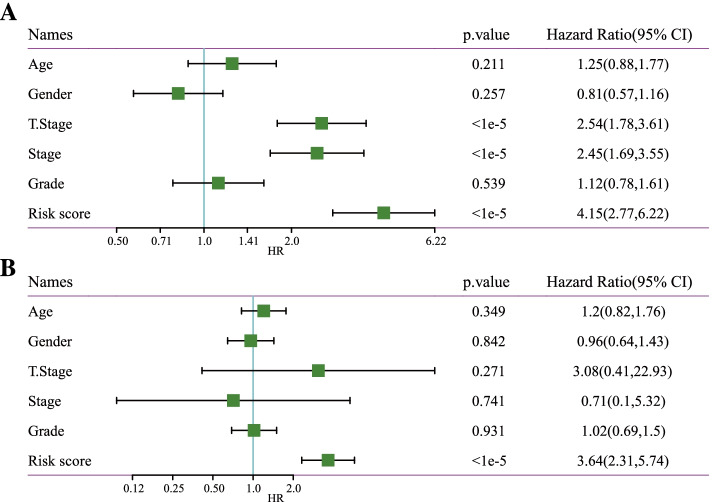
Univariate (**A**) and multivariate (**B**) Cox regression analysis for risk score and clinical features including age, gender, T stage, stage, and grade in TCGA-LIHC dataset. Log-rank test was performed. CI, confidence interval. HR, hazard ratio

In different subgroups, risk scores stratified by M stage, gender, age, N stage, American Joint Committee on Cancer (AJCC) stage, grade, T stage were compared. High-risk scored patients were more likely to be those with a more advanced T stage (*P* = 1.1e-05), and a high histological grade (*P* = 6e-07) and AJCC stage (*P* = 9.3e-06) in comparison with those showing low-risk scores (Fig. 4A). In addition, samples could be significantly stratified into the two risk groups of different clinical features, including gender (male and female), T stage (T1–2 and T3–4), age (age > 60 and age ≤ 60), AJCC stage (stage I-II and stage III-IV), and M stage (M0 stage), N stage (N0 stage) and grade (G1-G2 and G3-G4) (Fig. 4B). The LIHC samples showing a high-risk had generally significantly worse OS than low-risk patients, indicating that the risk model had strong predictive ability (Fig. 4B). Risk score, AJCC stage, T stage were noticeably associated with LIHC prognosis, as shown by univariate Cox regression analysis (Fig. 5A). After controlling confounding factors, multivariate Cox regression study demonstrated that the risk model was the only independent prognostic factor for LIHC (Fig. 5B).

### Tumor microenvironment and immune cells in each risk group

**Fig. 6:**
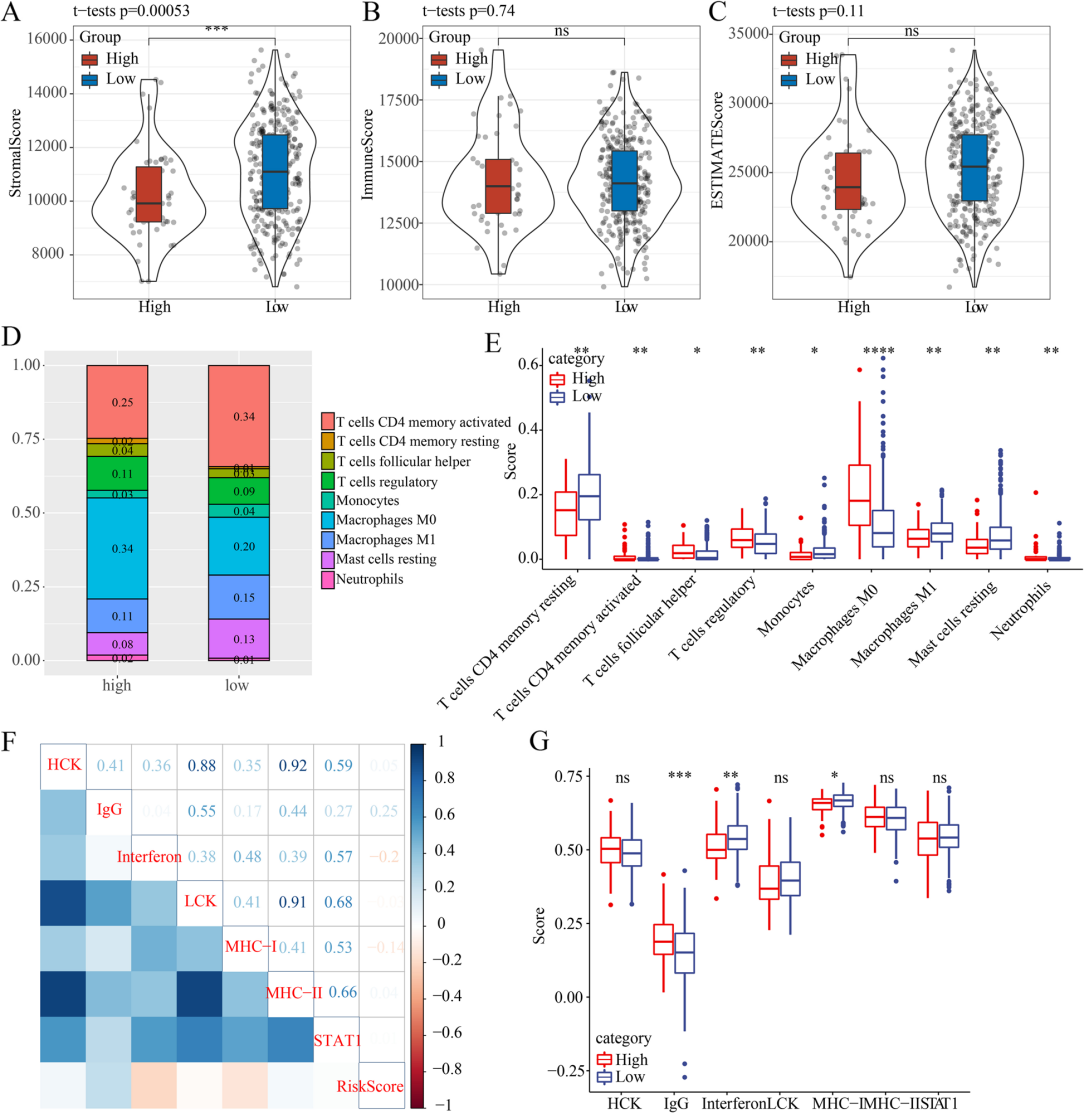
Evaluating the difference of immune features between low- and high-risk groups in TCGA-LIHC dataset. **A-C** Stromal score, immune score and ESTIMATE score of two risk groups. Student *t* test was conducted. **D** A barplot presenting the distribution of nine immune cell types with significant difference between two risk groups (*P* < 0.05). **E** Comparison of enrichment score of nine immune cell types between two risk groups. Student *t* test was conducted. **F** Pearson correlation analysis between risk score and ssGSEA score of nine metagenes. Red indicates negative correlation and blue indicates positive correlation. **G** SsGSEA score of seven metagenes in low- and high-risk groups. ns, no significance. *P < 0.05, **P < 0.01, ***P < 0.001, ****P < 0.0001

To better understand the immune microenvironment traits associated with the risk model developed based on 8 TGF-β pathway-related genes, the ESTIMATE, stromal and immune scores of each risk group were calculated by ESTAMATE. Stromal score was significantly lower in the high-risk group than low-risk group, while ESTIMATE score and immune score of the two risk groups showed no significant difference, suggesting that the high-risk group had a relatively low stromal component (Fig. 6A-C). Moreover, the difference analysis on the composition of 22 immune cells in the tumor microenvironment between the two risk groups demonstrated a significant difference in 9 immune cells (Fig. 6D, E). High-risk group showed a higher infiltration of helper follicular T cells, M0 macrophages, and regulatory T cells (Tregs), while resting memory CD4 T cells, monocytes, resting mast cells, M1 macrophages were more significantly enriched in low-risk group (Fig. 6E). Because of the linkage effect between cancer immunity and inflammation, 7 metagenes (including 105 genes related to inflammation and immune functions) was selected to calculate enrichment score by single sample gene set enrichment analysis (ssGSEA) in gene set variation analysis (GSVA) package. Risk score was negatively correlated with interferon and MHC- I (*R* = − 0.2 and − 0.14, respectively) but positively correlated with IgG (*R* = 0.25), as shown by correlation analysis (Fig. 6F). Although the correlations were not strong, a significant difference of ssGSEA score was shown on IgG, interferon, and MHC-I between the two risk groups in a boxplot (*P* < 0.05, Fig. 6G).

### Risk model based on 8 TGF-β pathway -related genes could predict the clinical response of immunotherapy/chemotherapy

**Fig. 7:**
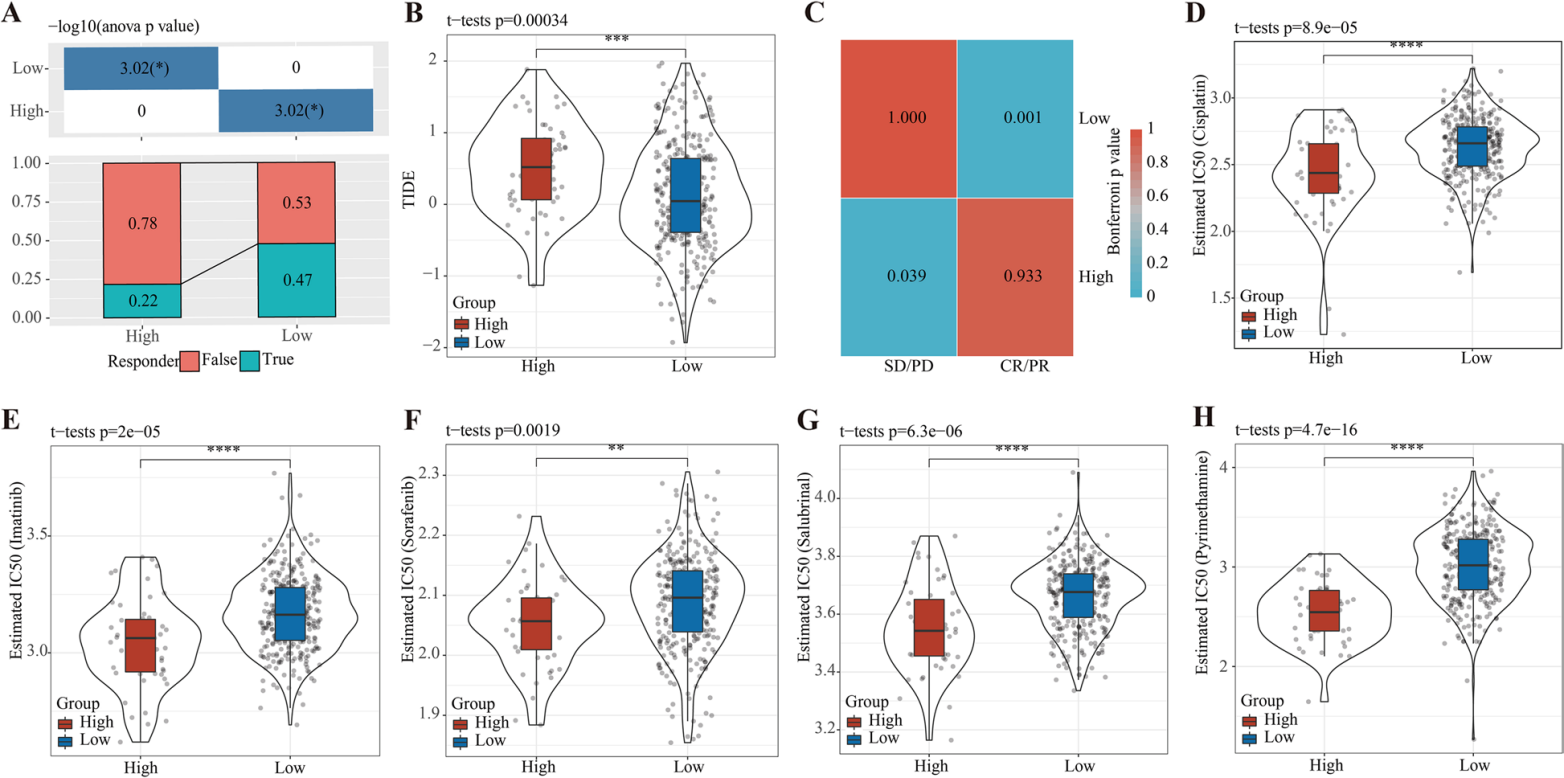
The predictive value of the prognostic model for immunotherapy and chemotherapy in TCGA-LIHC dataset. **A** The proportion of false and true responders in low- and high-risk groups analyzed by TIDE. False and true indicate non-responsive and responsive to immune checkpoint blockade. ANOVA test was performed, and *P* values transferred as –log10 (*P* value) were shown in the upper box. **B** Comparison of TIDE score between the two risk groups. Student *t* test was performed. **C** Submap analysis for analyzing the similarity between non-treated samples and anti-PD-1 treated samples. Bonferroni corrected *P* values were indicated in the box. **D-H** Estimated IC50 values of cisplatin (**D**), imatinib (**E**), sorafenib (**F**), salubrinal (**G**) and pyrimethamine (**H**) in two risk groups. Student *t* test was performed. PD, progressive disease. SD, stable disease. PR, partial response. CR, complete response. *P < 0.05, **P < 0.01, ***P < 0.001, ****P < 0.0001

Between the two risk groups, the difference in the score of infiltrating immune cell populations in tumor microenvironment indicated that the response of each risk group to immunotherapy required further exploration. The TIDE algorithm was utilized to analyze the response rate of immunotherapy of the two groups (Fig. 7A). Low-risk patients had significantly higher remission after taking immunotherapy than the high-risk. Moreover, TIDE score of low-risk patients was greatly lower (Fig. 7B). From the Submap analysis, it could be found that the low-risk group was more likely to respond to anti-PD-L1 treatment (Fig. 7C). The cisplatin, imatinib, sorafenib, salubrinal and pyrimethamine treatment-related IC50 in TCGA-LIHC samples was estimated to evaluate the sensitivity of the two risk groups to these drugs, and we found that high-risk LIHC samples were more sensitive to the above five drugs (Fig. 7D-H).

## Discussion

It is widely acknowledged that LIHC treatment is challenging for its high possibility of drug resistance. The development of clinically validated agents against LIHC has been significantly influenced by the complex interactions of liver tumors with their immune microenvironment and a lack of understanding of the heterogeneous mechanisms of LIHC tumorigenesis and progression [28]. Several studies have shown that dysregulated signals in the TGF-β pathway have important function in immune regulation in the LIHC microenvironment [8, 11]. Therefore, TGF-β pathway-targeted drugs, including drugs targeting TGF-β ligands, TGF-β receptors, and downstream mediators of TGF-β, have been explored and clinically tested. And all of those drugs can lead to a variety of synergistic downstream effects and may improve the clinical outcome of LIHC [29, 30]. At present, effective biomarkers should be discovered to help determine the response of tumor cells for LIHC patients [31].

This work developed a risk score model based on 8 TGF-β pathway-related genes through progressively screening 54 TGF-β pathway-related genes, which can score and group LIHC samples in independent datasets. Chen et al. found that about 40% of all LIHC samples showed at least one gene mutation in the TGF-β pathway [32]. In the high- and low-risk groups, TP53 was both identified to have the highest mutation frequency, which has been identified as a common molecular event in human liver cancer [33]. Previous studies have suggested that with the continuous acquisition of genomic mutations, tumor cells show a series of mutations in different signal pathways, resulting in changes in TGF- β response [34]. This also explained the function of TGF-β in the late stage of tumor was quite different from that in the early stage. In addition, GSEA showed that two risk groups had differential enriched pathways such as metabolism-related pathways like fatty acid metabolism, and drug metabolism were more enriched in low-risk group, which may be resulted from their differential mutation patterns in TP53 and metabolism-related genes.

A number of evidences show that TGF-β can modulate cellular responses that regulate the tumor microenvironment, which may also contribute to LIHC progression and drive immune escape of cancer cells [31]. On the comparison of tumor microenvironment and immune infiltration between two risk groups, we found that high-risk group had relatively higher enrichment of helper follicular T cells, Tregs and M0 macrophages. The previous study speculated that increased number of these immunosuppressive cells endowed high-risk group a strong immunosuppressive environment, leading to an unfavorable prognosis [34]. Importantly, two risk groups showed significantly different response to immunotherapy, where TIDE score of low-risk group was noticeably lower and responsive proportion was significantly higher, suggesting that low-risk group may be more responsive to anti-PD-L1 treatment. However, high-risk group was more sensitive to chemotherapeutic drugs or targeted drugs including cisplatin, imatinib, sorafenib and salubrinal and pyrimethamine. These observations suggested that the prognostic model had a potential in guiding immunotherapy or targeted therapy for LIHC patients.

Eight prognostic genes (CDKN1C, HDAC1, SERPINE1, BMP2, ENG, FKBP1A, NOG, and BCAR3) involved in TGF-β pathway were included in our prognostic model. We found that some of them were also identified as prognostic biomarkers for cancers by the previous studies. Cyclin-dependent kinase inhibitor 1C (CDKN1C, also known as p57(KIP2)), a tumor suppressor, could regulate tumor cell differentiation, invasion, and angiogenesis, which is also validated as a prognostic biomarker in various cancer types, including in LIHC [35, 36]. In a 7-gene hypoxia signature developed by Bai et al., CDKN1C has also been identified as a prognostic gene for predicting LIHC prognosis [37]. Histone deacetylase 1 (HDAC1) is a critical enzyme for epigenetic modification, whose overexpression is strongly correlated with tumor cell proliferation and growth in many cancers [38]. High expression of HDAC1 is significantly associated with elevated cancer-specific mortality in LIHC [39]. Plasminogen activator inhibitor 1 (SERPINE1, also known as PAI-1), is considered as a prognostic biomarker for gastric cancer, gliomas, and colorectal cancer [40–42], hepatocellular carcinoma [43]. SERPINE1 was also in an 8-gene prognostic by Lin et al [44] High expression of bone morphogenetic protein 2 (BMP2) could promote liver cancer cell growth through activating myeloid-derived suppressor cells [45]. Other four prognostic genes were less reported in LIHC research.

The 8-gene prognostic model manifested favorable performance in different datasets, except in GSE10143 dataset with an unsatisfied AUC. Nevertheless, our model still outperformed other present prognostic models for LIHC in the same datasets (TCGA-LIHC and ICGC). 1-year, 3-year and 5-year AUC were 0.76, 0.71 and 0.70 in TCGA-LIHC dataset, respectively. 1-year, 3-year and 4-year AUC were 0.76, 0.75 and 0.64 in ICGC dataset, respectively. We included some studies containing at least one prognostic gene as our model. Sun et al. established a 2-gene prognostic model (CANX and HDAC1) for LIHC based on immune-related and autophagy-related genes using TCGA-LIHC and ICGC datasets [46]. The AUC of the 2-gene prognostic model for 1-year, 3-year and 5-year was 0.696, 0.639 and 0.642 in TCGA training dataset, 0.728, 0.685 and 0.612 in TCGA test dataset, 0.757, 0.669 and 0.644 in ICGC dataset, respectively. Lin et al. constructed an 8-gene prognostic model (SLC7A1, RIPK2, NOD2, ADORA2B, MEP1A, ITGA5, P2RX4, and SERPINE1) based on inflammatory response-related genes for LIHC also utilizing TCGA-LIHC and ICGC datasets [44]. The AUC of Lin et al’s model for predicting 3-year OS was 0.614 and 0.710 in TCGA-LIHC and ICGC datasets, respectively [44]. Compared with other prognostic models, our model was validated in more datasets, while they only validated their models in ICGC dataset.

This study had some limitations. Firstly, all the data were retrospective data, and experiments were not designed to verify them from other aspects. Secondly, our analysis was only based on TGF-β pathway-related genes, and the results did not represent all LIHC-related gene profiles. Thirdly, algorithms for characterizing tumor microenvironment, such as ESTIMATE and CIBERSORT, are not always accurate due to the atypical or unclear tumor microenvironment varying by tumor types. There was a possibility that an overlap of some gene signatures may exist between stromal cells and tumor cells because of the influence of epithelial-to-mesenchymal transition (EMT). In the future, the scope of research should be further expanded and experimental studies should be carried out to analyze the risk model based on TGF-β pathway-related genes on LIHC pathological behavior.

## Conclusions

In conclusion, a risk score model with stepwise analysis of 54 TGF-β pathway-related genes in TCGA-LIHC was developed, which can independently predict the LIHC prognosis and was related to the response to immunotherapy or chemotherapy, immune cell infiltration, tumor microenvironment. This study provided a perspective to elucidate LIHC clinical outcomes from the perspective of TGF-β pathway-related genes, offering novel possibility for improving LIHC management.

## Supplementary Information

**Additional file 1: Supplementary Table S1.** A list of 54 TGF-β pathway-related genes.

**Additional file 2: Supplementary Table S2.** Clinical features of the LIHC samples in different datasets.

**Additional file 3: Supplementary Table S3.** Classification of low- and high-risk groups in five datasets.

## Data Availability

The datasets generated during and/or analysed during the current study are available in the [GSE14520] repository, [https://www.ncbi.nlm.nih.gov/geo/query/acc.cgi?acc=GSE14520]; in [GSE76427] repository, [https://www.ncbi.nlm.nih.gov/geo/query/acc.cgi?acc=GSE76427]; in [GSE10143] repository, [https://www.ncbi.nlm.nih.gov/geo/query/acc.cgi?acc=GSE10143].
