# Supplementary material for: A risk score model based on TGF-β pathway-related genes predicts survival, tumor microenvironment and immunotherapy for liver hepatocellular carcinoma
Source: Proteome Sci. 2022 Jun 22;20:11. doi: 10.1186/s12953-022-00192-4 (PMC9215003; doi:10.1186/s12953-022-00192-4)
Supplement: Supplementary file 1 — Additional file 1: Supplementary Table S1. A list of 54 TGF-β pathway-related genes. [file 12953_2022_192_MOESM1_ESM.docx]

Supplementary Table S1. A list of 54 TGF-β pathway-related genes.

| TGFBR1 |
| --- |
| SMAD7 |
| TGFB1 |
| SMURF2 |
| SMURF1 |
| BMPR2 |
| SKIL |
| SKI |
| ACVR1 |
| PMEPA1 |
| NCOR2 |
| SERPINE1 |
| JUNB |
| SMAD1 |
| SMAD6 |
| PPP1R15A |
| TGIF1 |
| FURIN |
| SMAD3 |
| FKBP1A |
| MAP3K7 |
| BMPR1A |
| CTNNB1 |
| HIPK2 |
| KLF10 |
| BMP2 |
| ENG |
| APC |
| PPM1A |
| XIAP |
| CDH1 |
| ID1 |
| LEFTY2 |
| CDKN1C |
| TRIM33 |
| RAB31 |
| TJP1 |
| SLC20A1 |
| CDK9 |
| ID3 |
| NOG |
| ARID4B |
| IFNGR2 |
| ID2 |
| PPP1CA |
| SPTBN1 |
| WWTR1 |
| BCAR3 |
| THBS1 |
| FNTA |
| HDAC1 |
| UBE2D3 |
| LTBP2 |
| RHOA |
