# Supplementary material for: A risk score model based on TGF-β pathway-related genes predicts survival, tumor microenvironment and immunotherapy for liver hepatocellular carcinoma
Source: Proteome Sci. 2022 Jun 22;20:11. doi: 10.1186/s12953-022-00192-4 (PMC9215003; doi:10.1186/s12953-022-00192-4)
Supplement: Supplementary file 2 — Additional file 2: Supplementary Table S2. Clinical features of the LIHC samples in different datasets. [file 12953_2022_192_MOESM2_ESM.docx]

| **Clinical Features** | **TCGA-LIHC** | **GSE10143** | **GSE14520** | **GSE76427** | **ICGC** |
| --- | --- | --- | --- | --- | --- |
| **Total samples** | 365 | 80 | 221 | 115 | 203 |
| **OS** |  |  |  |  |  |
| 0 (alive) | 235 | 48 | 136 | 92 | 168 |
| 1 (dead) | 130 | 32 | 85 | 23 | 35 |
| **T Stage** |  |  |  |  |  |
| T1 | 180 | / | / | / | 33 |
| T2 | 91 | / | / | / | 96 |
| T3 | 78 | / | / | / | 59 |
| T4 | 13 | / | / | / | 15 |
| TX | 3 | / | / | / | / |
| **N Stage** |  |  |  |  |  |
| N0 | 248 | / | / | / | / |
| N1 | 4 | / | / | / | / |
| NX | 113 | / | / | / | / |
| **M Stage** |  |  |  |  |  |
| M0 | 263 | / | / | / | / |
| M1 | 3 | / | / | / | / |
| MX | 99 | / | / | / | / |
| **Stage** |  |  |  |  |  |
| Ⅰ | 170 | / | 93 | 55 | / |
| Ⅱ | 84 | / | 77 | 35 | / |
| III | 83 | / | 49 | 21 | / |
| Ⅳ | 4 | / | / | 3 | / |
| X | 24 | / | 2 | 1 | / |
| **Grade** |  |  |  |  |  |
| G1 | 55 | / | / | / | / |
| G2 | 175 | / | / | / | / |
| G3 | 118 | / | / | / | / |
| G4 | 12 | / | / | / | / |
| GX | 5 | / | / | / | / |
| **Gender** |  |  |  |  |  |
| Male | 246 | / | 191 | 93 | 153 |
| Female | 119 | / | 30 | 22 | 50 |
| **Age** |  |  |  |  |  |
| ≤ 60 | 173 | / | / | 48 | 43 |
| ＞ 60 | 192 | / | / | 67 | 160 |

**Supplementary Table S2. Clinical features of the LIHC samples in different datasets.**

/ indicates unknown data. In OS part, 0 indicates alive samples (not censored) and 1 indicates dead samples (censored).
