# Supplementary material for: A risk score model based on TGF-β pathway-related genes predicts survival, tumor microenvironment and immunotherapy for liver hepatocellular carcinoma
Source: Proteome Sci. 2022 Jun 22;20:11. doi: 10.1186/s12953-022-00192-4 (PMC9215003; doi:10.1186/s12953-022-00192-4)
Supplement: Supplementary file 3 — Additional file 3: Supplementary Table S3. Classification of low- and high-risk groups in five datasets. [file 12953_2022_192_MOESM3_ESM.docx]

Supplementary Table S3. Classification of low- and high-risk groups in five datasets.

| Datasets | Optimal cut-off values | Number of samples in low-risk group | Number of samples in high-risk group |
| --- | --- | --- | --- |
| TCGA-LIHC | 3.924 | 314 | 51 |
| GSE14520 | 5.053 | 188 | 33 |
| GSE76427 | -0.727 | 14 | 101 |
| GSE10143 | -16.180 | 27 | 53 |
| ICGC | 3.257 | 166 | 37 |
